# Erectile dysfunction in men with chronic obstructive pulmonary disease: cross-sectional study in a Spanish population

**DOI:** 10.1590/1806-9282.20252266

**Published:** 2026-08-07

**Authors:** Nicolás Alcalá-Rivera, Jesús Díez-Manglano

**Affiliations:** 1 Barbastro Hospital, Department of Internal Medicine – Huesca, Spain.; 2 Hospital Royo Villanova, Department of Internal Medicine – Zaragoza, Spain.; 3 Barbastro Hospital – Huesca, Spain.

**Keywords:** Erectile dysfunction, Chronic obstructive pulmonary disease, Male, Sexuality

## Abstract

**OBJECTIVE:**

The aim of this study was to describe sexual life and determine the prevalence of erectile dysfunction in men with chronic obstructive pulmonary disease.

**METHODS:**

A multicenter cross-sectional study was conducted with 120 male patients diagnosed with chronic obstructive pulmonary disease for 2 years since 2021. Participants completed a comprehensive questionnaire that included the International Index of Erectile Function, selected items from the Questionnaire on Changes in Sexual Function, and additional questions about sexual health. Erectile dysfunction was considered clinically significant with an International Index of Erectile Function score ≤16. No formal a priori sample size calculation was performed because participant recruitment was based on consecutive inclusion during a 2-year study period.

**RESULTS:**

In total, 113 participants (94.2%) reported experiencing changes in their sex life and 42 (36%) were dissatisfied. The main factors contributing to these changes were the underlying disease (53.3%), medication use (20.8%), and psychological problems (20%). The mean International Index of Erectile Function score was 13.8 (7.0), with an erectile dysfunction prevalence of 87.5%. Erectile dysfunction was mild in 33 patients (27.5%), mild-to-moderate in 26 (21.7%), moderate in 21 (17.5%), and severe in 25 (20.8%). Multivariate analysis identified dyslipidemia (odds ratio: 4.028; 95%CI 1.428–11.361; p=0.008) and %pFEV1 (OR 0.937; 95%CI 0.906–0.968; p<0.001) as independent predictors of clinically significant erectile dysfunction.

**CONCLUSION:**

Poor sexual health and erectile dysfunction are highly prevalent among men with chronic obstructive pulmonary disease. Dyslipidemia and impaired airflow are associated with the presence of clinically significant erectile dysfunction.

## INTRODUCTION

Chronic obstructive pulmonary disease (COPD) is a progressive disease characterized by airflow limitation that is not fully reversible. Current estimates indicate that approximately 56 million people in Europe suffer from COPD, with a global mortality rate of around 3 million annually^1,2^. The most prevalent symptoms of COPD, such as cough, dyspnea, fatigue, and pain, significantly affect patients’ physical and psychological well-being, leading to a marked reduction in their quality of life^3^. In addition, exercise intolerance, anxiety, depression, and hypoxemia may negatively affect sexual activity and satisfaction.

Patients with COPD frequently present with comorbidities that play a fundamental role in the progression and prognosis of the disease^4^. These comorbidities, along with other extrapulmonary manifestations such as heart failure, diabetes mellitus, peripheral arterial disease, musculoskeletal dysfunction, osteoporosis, or depression contribute to a substantial deterioration in quality of life. Furthermore, these factors are associated with a higher risk of disease exacerbations and subsequent hospitalizations^5^.

Sexual health is a fundamental aspect of overall well-being. Chronic diseases frequently have a negative impact on sexual health, resulting in a decreased quality of sexual life. Recent studies indicate that more than 50% of men and women with COPD experience sexual dysfunction^6,7^. However, the relationship between COPD and sexual activity remains underexplored, and the specific factors contributing to sexual dysfunction in this population are not yet fully understood. Furthermore, it’s an aspect infrequently addressed in COPD management in routine clinical practice.

The primary objective of this study was to assess the sexual lives of men with COPD. Secondary objectives included determining the prevalence of ED among these patients and identifying clinical and pulmonary function differences between COPD patients with and without ED.

## PATIENTS AND METHODS

### Study design

This was a cross-sectional, descriptive, multicenter study involving patients from two hospitals and several primary care centers in Aragon, Spain. The study was approved by the Ethics Committee of the Aragon Clinical Research (CEICA) with file number PI 20/348, and all participants received their written consent.

### Inclusion and exclusion criteria

The study population consisted of men over 40 years of age with a confirmed diagnosis of COPD during 2 years. The COPD diagnosis was based on spirometry performed under stable conditions, with the evaluation carried out within the last 2 years, according to GOLD guidelines^8^. Exclusion criteria included illiterate patients, those who refused to participate, and individuals over 85 years of age.

### Variables and data collection

Patients were included who were admitted to our hospital through the registration of diagnoses of the internal health system itself, and the search for patients in health centers through recruitment by various consultations of primary care family and community medicine doctors. They were administered a comprehensive questionnaire upon admission or during outpatient visits. This questionnaire included relevant demographic and clinical data, the 5-item version of the IIEF-5^9^, selected items from the Changes in Sexual Function Questionnaire (CSFQ)^10^, and additional questions related to sexual health. The data collected included age, body mass index (BMI), smoking history, alcohol consumption, and medication use. Comorbidities were assessed using the age-adjusted Charlson Comorbidity Index^11^, supplemented with other conditions prevalent in COPD, such as hypertension and dyslipidemia. BMI was calculated by dividing weight by the square of height and expressed in kg/m^2^. ED was assessed using the IIEF-5, which consists of five Likert-type questions and provides a score from 5 to 25, where lower scores indicate worse ED^9^. ED was classified as absent, mild, mild to moderate, moderate, or severe, with scores of 22–25, 17–21, 12–16, 8–11, and 5–7, respectively. Deficiency was classified as clinically significant with an IIEF-5 score ≤16. Written informed consent was obtained from all participants.

### Statistical analysis

The normality of continuous quantitative variables was assessed using the Kolmogorov-Smirnov test. Quantitative data are presented as mean (standard deviation) for normally distributed variables and as median (interquartile range) for non-normally distributed variables. Comparisons between groups were performed using the Student’s t-test or the Mann-Whitney U test, as appropriate. Qualitative data are presented as absolute frequencies and percentages, and comparisons between two groups were performed using the chi-square test or Fisher’s exact test.

The correlation between FEV1 and the IIEF-5 score was assessed using Pearson’s correlation coefficient. A logistic regression model was developed incorporating the variables that showed statistical significance in the univariate analysis. The goodness of fit of the logistic regression model was assessed using the Hosmer-Lemeshow test. A p<0.05 was considered statistically significant in all analyses. Statistical analyses were performed using SPSS software, version 22.0, for Windows.

## RESULTS

A total of 120 men were included in the study, 15 from primary care centers and 105 from hospitals. The mean age of the participants was 65.6 (9.7) years. Patient characteristics are presented in Table 1.

**Table 1:** General clinical characteristics, spirometric data and relevant medical information.

	Total (n=120)	No ED or mild ED (n=48)	Other degrees of ED (n=72)	p-value
Age	65.5 (±9.6)	59.7 (±8.2)	69.4 (±8.6)	<0.001
BMI	29.2 (±5.5)	28.3 (±6.4)	29.8 (±4.9)	0.189
Smoking				
Smoker	21 (17.5%)	12 (25%)	9 (12.5%)	0.220
Former smoker	98 (81.7%)	36 (75%)	62 (86.1%)	
Passive smoker	1 (0.8%)	0	1 (1.4%)	
Alcohol consumption	49 (40.8%)	25 (52.1%)	24 (33.3%)	0.044
Taking antidepressants	11 (9.2%)	5 (10%)	6 (8.3%)	0.707
Package-year index	48.8 (±34)	38.8 (±9.4)	55.5 (±42.3)	0.002
FEV1	58 (±19.4)	68.8 (±15.4)	50.9 (±18.6)	<0.001
FEV1/FVC	58 (±10)	58.2 (±12.1)	58.3 (±8.4)	0.978
Degree of obstruction in spirometry				
1	23 (19.2%)	22 (45.8%)	1 (1.4%)	<0.001
2	66 (55%)	21 (43.8%)	45 (62.5%)	
3	12 (10%)	3 (6.3%)	9 (12.5%)	
4	19 (15.8%)	2 (4.2%)	17 (23.6%)	
GOLD classification				
A	33 (27.5%)	26 (54.2%)	7 (9.7%)	<0.001
B	56 (46.7%)	20 (41.7%)	36 (50%)	
C	17 (14.2%)	2 (4.2%)	15 (20.8%)	
D	14 (11.7%)	0 (0.0%)	14 (19.4%)	
Diabetes mellitus type 2	31 (25.8%)	28 (39.6%)	12 (16.7%)	0.008
Arterial hypertension	76 (63.3%)	28 (58.3%)	48 (66.7%)	0.363
Heart failure	34 (28.3%)	10 (20.8%)	24 (33.3%)	0.128
Chronic ischemic heart disease	21 (17.5%)	9 (18.8%)	12 (16.7%)	0.773
Peripheral vascular disease	11 (9.2%)	2 (4.2%)	6 (8.3%)	0.091
Dyslipidemia	51 (42.5%)	15 (31.3%)	36 (50%)	0.040
Renal insufficiency	2 (1.7%)	48 (100%)	2 (2.8%)	0.159
Charlson index corrected for age	4.8 (±1.5)	4.4 (±1.5)	5 (±1.5)	0.029
Inhalers				
Monotherapy	10 (8.4%)	7 (14.6%)	3 (4.2%)	<0.001
Double therapy	67 (55.8%)	38 (79.2%)	29 (40.3%)	
Triple therapy	43 (35.8%)	3 (6.3%)	40 (55.6%)	
IIEF-5	13.7 (±6.9)	20.8 (±1.9)	9 (±4.8)	<0.001

The variables are expressed as mean and standard deviation or n (%). ED: erectile dysfunction; BMI: body mass index; FEV1: forced expiratory volume in 1 second; FEV1/FVC: percentage of forced vital capacity that is expired in the first second; IEF-5: International Index of Erectile Function.

### Chronic obstructive pulmonary disease and sexual life

Of the participants, 114 identified as heterosexual, four as bisexual, and two as homosexual. Notably, 7 men (5.8%) and 24 men (20.0%) reported that their sexual lives were not very pleasurable or not pleasurable, respectively. In addition, 89 men (74.2%) had infrequent sexual activity, defined as once a month or less. The majority of patients (94.2%) had experienced a change or decrease in their sexual life, which they attributed to the underlying disease (53.3%), medications (20.8%), psychological problems (20.0%), age (7.5%), or lack of a partner (7.5%). In total, 99 people believed that COPD affected their sex life.

During sexual activity, 78 patients experienced dyspnea: 61 (50.8%) attributed it to COPD, while 17 (14.2%) attributed it to other causes. In addition, 25 (20.8%) required the use of inhalers. Notably, 21 men reported infrequent ejaculation, defined as once a month or less. In total, 52 (43.3%) patients reported no sexual satisfaction or very infrequent sexual satisfaction.

### Prevalence of erectile dysfunction

The mean IIEF-5 score was 13.8 (7.0). Erectile dysfunction (ED) was observed in 105 men (87.5%). The severity of ED was classified as mild in 33 patients (27.5%), mild-to-moderate in 26 patients (21.7%), moderate in 21 patients (17.5%), and severe in 25 patients (20.8%). In total, 72 patients (60.0%) reported clinically significant ED and 46 patients were unable to achieve or maintain an erection. These patients were older, had more comorbidities, particularly diabetes and dyslipidemia, and experienced more severe airflow obstruction (Table 1). Patients with non-mild ED were significantly older and had a higher cumulative smoking index (CSI) (p=0.002). They also showed worse lung function, with significantly lower FEV1 values (p<0.001), although no difference was observed in the FEV1/FVC ratio. Regarding the severity of obstruction, patients with non-mild ED were significantly more prevalent (p<0.001), with a higher proportion in severe stages (grade 3–4 obstruction), while patients without ED or with mild ED were mainly concentrated in milder stages. Consistently, the GOLD classification showed significant differences (p<0.001), with a clear trend toward a higher prevalence of non-mild ED in the more severe groups, especially in GOLD E (96%). In the multivariate analysis, dyslipidemia (OR 4.028, 95%CI 1.428–11.361; p=0.008) and postoperative FEV1 percentage (OR 0.937, 95%CI 0.906–0.968; p<0.001) were independently associated with clinically significant ED (Table 2).

**Table 2:** Logistic regression analysis comparing patients with no or mild erectile dysfunction with the rest.

	Univariate		Multivariate	
	OR (95%CI)	p-value	OR (95%CI)	p-value
Alcohol consumption	0.460 (0.218–0.973)	0.042	0.425 (0.166–1.086)	0.074
Smoking (package-year index)	1.027 (1.004–1.051)	0.021	1.022 (0.998–1.047)	0.075
VEF1	0.940 (0.916–0.966)	<0.001	0.937 (0.906–0.968)	<0.001
Dyslipidemia	2.200 (1.023–4.730)	0.044	4.028 (1.428–11.361)	0.008
Charlson index corrected for age	1.311 (1.023–1.681)	0.032	1.086 (0.798–1.478)	0.601

CI: confidence interval; OR: odds ratio; FEV1: forced expiratory volume in the first second.

## DISCUSSION

The main findings of our study indicate that sexual life is frequently unsatisfactory and that ED is highly prevalent among men with COPD, affecting almost 9 out of 10 patients. Furthermore, ED is associated with dyslipidemia and a more severe degree of airway obstruction.

A study conducted in France demonstrated that 4 out of 10 patients with COPD were not sexually active, and among those who remained sexually active, 60% had to modify their sex life due to the disease^12^. Similarly, in our study, less than half of the patients were satisfied with their sex life.

Half of the patients reported experiencing dyspnea during sexual activity and attributed it to COPD. Less than 10% regularly use inhaler therapy during such activity. Asking patients whether they use their inhalers before, during, or immediately after sexual activity could initiate a dialogue about their sex lives and address this important aspect of well-being. The use of informational brochures may also be helpful.

Previous studies conducted outside of Spain have reported a prevalence of ED exceeding 70% in patients with COPD^13,14^. The exact mechanism of ED remains unknown, but there is a consensus that a vasculogenic deficit occurs during the progression of certain chronic diseases, such as hypertension, diabetes, renal failure, depression, or anxiety. The end result is a reduction in serum testosterone levels, which contributes to muscle wasting^15,16^. The use of certain medications has also been linked to the development of ED. In the case of COPD, the causes of ED are not yet fully understood, but several potential risk factors have been proposed. Endothelial dysfunction due to hypoxemia.

Within the corpora cavernosa, decreased oxygen levels lead to reduced production of prostaglandin E1, which inhibits profibrotic cytokines. Increased levels of these cytokines result in the replacement of smooth muscle with collagen, impairing compression of the sub-tunical veins and ultimately, erection^17^. Other mechanisms, including chronic systemic inflammation, reduced nitric oxide levels, decreased testosterone levels, aging, psychosocial problems, and reduced physical activity, are also thought to contribute to the development of impotence^18^.

Our findings reveal differences in the factors associated with ED. Turan et al.^19^ reported a negative correlation between pack-years and IIEF scores. While we observed a higher smoking burden among men with clinically significant ED, our multivariate analysis did not identify an independent association. Nevertheless, the relationship between smoking intensity and duration and ED is well-established in the general population^18,20^. Most men with ED in our study were classified as GOLD phenotype C or D, which is consistent with a study conducted in Portugal that also reported a higher incidence of ED at these stages^15^. This implies that patients who experience frequent acute exacerbations may be at increased risk of developing clinically significant ED. Similarly, Kahraman et al. identified a negative correlation between the degree of airway obstruction, as measured by FEV1, and ED, a finding consistent with our results and those of other studies^14^. This suggests that a greater degree of airway obstruction may lead to increased hypoxia and vascular deterioration, ultimately contributing to the development of ED. It is well-established that cardiovascular disease can cause ED and that COPD is associated with increased cardiovascular risk, which increases with the severity of airway obstruction^21^. This relationship could explain the higher prevalence of ED among patients with more severe COPD.

In our study, we identified an association between dyslipidemia and ED. Dyslipidemia has also been linked to the development of ED in patients with other chronic diseases, such as diabetes^17^. Furthermore, a meta-analysis has demonstrated that statin therapy can have a beneficial effect on erectile function^22^.

Our study has several limitations that should be acknowledged. First, the use of sexual health questionnaires can introduce recall and social desirability biases. However, it is important to note that the IIEF-5 and the CSFQ are globally validated instruments widely used in diverse populations and disease groups. Second, hormonal assessments that could help to better characterize sexual function were not performed. Third, most participants were recruited in a hospital setting, which could result in selection bias and may limit the applicability of our findings to community-dwelling patients. Finally, the cross-sectional design of our study may not reflect changes in sexual function over time, which could vary depending on disease progression, treatments, seasonality, or personal circumstances.

## CONCLUSION

Sexual health is significantly affected in men with COPD, with ED being a highly prevalent condition. The severity of ED may correlate with the severity of airflow obstruction and the presence of dyslipidemia. It is essential to consider this crucial aspect of quality of life in the management of these patients, and further research is needed to better understand this issue.

## Data Availability

The datasets generated and/or analyzed during the current study are available from the corresponding author upon reasonable request.
